# Supplementary material for: An exploratory study on the impact of ESG on business performance—Focusing on listed companies in Korea and Taiwan
Source: PLoS One. 2024 Nov 22;19(11):e0310447. doi: 10.1371/journal.pone.0310447 (PMC11584088; doi:10.1371/journal.pone.0310447)
Supplement: Attachment — Submitted filename: Response to Reviewers.docx [file pone.0310447.s004.docx]

I sincerely thank the editor and al reviewers fortheir valuable feedback that Ihave used to improve the quality of our manuscript.The reviewer comments are laid out below in italicized font and specific concerns have been numbered.My response is given in normal font and changes/additions to the manuscript are given in the blue text.

*Reviewer #1：*

*It was very interesting reading this paper. Thank you for including me as reviewer. The paper provides a comprehensive analysis of ESG practices and their impact on business performance in Korea and Taiwan. The comparative approach between these two regions, considering their unique economic structures, is particularly insightful and adds significant value to the existing literature on ESG. As I am from Europe, it was very interesting for me to read it but also to know a lot of new things from this part of world. The use of empirical data from the Bloomberg database and the application of Tobin's q ratio as a measure of business performance are commendable methodological choices by my opinion. Overall, this paper makes a significant contribution to the field of ESG and business performance. With some minor enhancements, it has the potential to offer even deeper insights and broader implications for academia and industry practitioners. I encourage researchers to continue in this direction.*

I feel great thanks for your professional review work on our article. As you are concerned, there are several problems that need to be addressed.According to your nice suggestions, we have made extensive corrections to our previous draft.The specific corrections are as follows:

1.I feel sorry for our carelessness.In my resubmitted manuscript,the typo is revised. Thanks for your correction.

2.I tried my best to improve the manuscript and made some changes to the manuscript. These changes will not influence the content and framework of the paper.

3.We sincerely appreciate the valuable comments. Wehave checked the literature carefully and added more references on Digital transformation and Model stability in the revised manuscript.

4. Some data errors were corrected, and the instability of the variable of advertising input was explained and explained.

*Reviewer #2：*

*1. In Table 2 (Descriptive statistics), the minimum value for Debt ratio is 0.000. What percentage of your sample is all-equity? (Some of your metrics have 3 decimal places, others have 2 or 1, please make them uniform, in the US 2 is standard).*

The company with a debt ratio of 0 is STUDIO DRAGON CORP of South Korea, code 253450.

Unified decimals, the article unified into 3 decimals. The reason is that some data is too small, if it is two decimals, some data is 0.00, three decimals are more accurate.

*2. In Table 4 (Model Regression Results), the 95% confidence interval for the Equity variable is incorrectly reported as "-3.758 -0.001". This doesn't match the positive coefficient (0.0503) and p-value (0.002) reported for this variable. Please address this. It's likely a typo. (Some of your metrics have 3 decimal places, others have 4, please make them uniform, in the US 2 is standard).*

This problem does exist because of a typo, because Debt and Equity are adjacent lines, and later in the writing process, 95% of these two questions were incorrectly confconfed. Written for the same, now modified. Thank you for your careful review!

*3. In Tables 5 and 6 (regression results for Korean and Taiwanese firms), different sets of variables are included without explanation. For example, ROA is included in Table 4 but excluded in T5,6. Given that ROE varies widely for Taiwanese firms vs Korean firms, perhaps it could be included (or a brief reason provided, thank you). Also the Titles for T5 and T6 should be very similar, to convey it is a comparative panel table. Pls. standardize decimal length in these tables too. The technological footprint of Kr and Tw could be one of the factors for this ROE divergence. The significance of technology is well highlighted in Dionisio, Marcelo,et al. "Role of digital transformation in improving the efficacy ..."Journal of High Technology Management Research (2023).*

The regression data of some variables in Table 4 and Table 5 are supplemented to make the article more complete. However, ROA was not included in the regression model constructed in this paper. According to Dionisio, Marcelo,et al (2023), the paper explained why ROA was not included in the model, and carried out scalability analysis.

*4. In Table 7 (Regression Analysis Results of ESG Sub-dimensions), the coefficient for ROE is negative (-0.191), which seems to contradict the positive coefficient (0.045) reported for ROE in Table 4. Also a formal model using equation editor should be listed for T5,6,7 with Tobin's Q as the Dependent variable and others as the X variables.*

The models for Tables 5 and 6 are written out above Tables 5 and 6.

The model for Table 7 is written out above Table 7.

In the regression analysis of each subdimension, the regression results of growth, size, ROE and R&D were corrected. This is really the author's oversight, thank you for pointing out the shortcomings of this article, based on your comments, I believe the quality of the article will become better

*5. In Table 7, the coefficient for Advertising (0.083) is positive, somewhat contradicting the negative coefficients reported for this variable in previous tables. Or please elaborate on it briefly. It is possible there is factor instability (which is fine), Agrrawal and Clark (2009), attribute factor/coefficient instability to variance discontinuities and note their impact on liquidity of assets "Multivariate Liquidity Score and Ranking Device for ETFs." Academy of Financial Services (2009).*

The correlation between advertising investment and Tobin Q of Korean enterprises and Taiwan enterprises is negative, but in the regression of the fractal dimension, the results can be significantly different from each other. Therefore, this paper has listened to your ideas and combined agrawal and Clark(2009) to explain the reasons for the instability of the coefficient. This will improve the quality of the article.

I appreciate the reviewers' thorough evaluation and recognize the major concerns raised regarding the clarity of my main argument and the completeness of the theoretical framework.I sincerely appreciate the time and effort invested by the reviewers in evaluating our manuscript. I look forward to any additional feedback or suggestions.

Sincerely，

Corresponding author FAN SHAOJIE
